# Genome‐Wide SNP Data and Species Distribution Modeling Reveal Population Structure and Conservation Implications of *Primula wilsonii* (Primulaceae)

**DOI:** 10.1002/ece3.72884

**Published:** 2026-01-05

**Authors:** Yanping Xie, Xin Jin, Ganggang Yang, Dongting Lu, Chan Zhang, Xingwang Zhang, Xianfeng Jiang

**Affiliations:** ^1^ School of Life Sciences Huaibei Normal University Huaibei China; ^2^ School of Life Sciences Henan Normal University Xinxiang China; ^3^ College of Agriculture and Bioscience Dali University Dali China

**Keywords:** ddRAD‐seq, demographic history, genetic diversity, population genetic structure, SNP, species distribution model (SDM)

## Abstract

Integrating population genetics with species distribution models provides powerful insights into species' evolutionary trajectories under climatic and geological contexts, and informs evidence‐based conservation strategies for endangered species. In this study, we characterized the population genetic structure of *Primula wilsonii* , a plant species with extremely small populations (PSESP) endemic to the mountains of Southwest China, to provide insights for the conservation of alpine endangered plants. Double‐digest restriction‐site associated DNA sequencing (ddRAD‐seq) generated 18,313 neutral and unlinked single‐nucleotide polymorphisms (SNPs) from 30 individuals across seven populations. Population genetic analysis revealed that these populations formed two major clusters. All populations exhibited low levels of genetic diversity (*He* = 0.00184–0.00271; π = 0.00176–0.00292), with one population (MG1) showing high genetic differentiation (*F*_ST_ > 0.7) from the other populations, possibly reflecting long‐term geographic isolation resulting in a small effective population size (*Ne*). Demographic history analysis indicated an expansion around 0.88 Ma, followed by sustained contraction since approximately 45 ka with low *Ne* levels maintained. Species distribution models further suggested range contraction from the Last Glacial Maximum to the mid‐Holocene and persistence of suitable refugia in the Hengduan Mountains under future warming scenarios. In conclusion, our results indicated that complex topography and Quaternary climatic oscillations have shaped a hierarchical genetic structure with deeply isolated lineages. We therefore recommend delineating at least three independent management units, consistent with patterns of genetic structure, differentiation, and demographic history. These findings highlight the importance of integrating genetic and environmental evidence in the conservation of *P. wilsonii* and other PSESPs.

## Graphical abstract

This study investigates the genetic erosion and conservation of *Primula wilsonii* , a plant species with extremely small populations in Southwest China. Using genomic sequencing and species distribution models, the research identifies distinct genetic clusters, low genetic diversity, and significant genetic differentiation among populations, in the text of complex topography and climatic oscillations. The findings highlight the need for targeted conservation efforts.

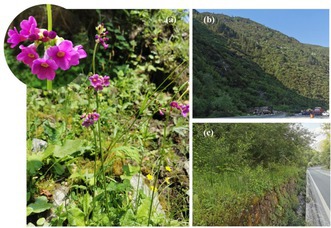

## 1 Introduction

Biodiversity is a fundamental component of the Earth's life‐support system, encompassing species diversity, ecosystem diversity, and genetic diversity. However, habitat loss and fragmentation, overexploitation, environmental pollution, biological invasions, and climate change have placed more than one million species at risk of extinction (Mouillot et al. 2013; De Vos et al. 2015). This accelerating erosion of biodiversity underscores the urgent need to understand and preserve genetic variation, particularly in narrowly distributed and vulnerable plant species. With rapid population growth and economic development, China faces increasing challenges in biodiversity conservation driven by long‐term human activities. Approximately 18.83% of higher plant species are threatened, near‐threatened or facing extinction, highlighting the urgency of plant diversity conservation in China (Ren et al. 2012; Qin et al. 2017; Zang 2020). Among these, plant species with extremely small populations (PSESP) typically exhibit narrow distributions and are subject to long‐term external disturbances. Such species often fall below the minimum viable population threshold for stable survival and therefore remain at a high risk of extinction, necessitating urgent need for conservation (Ren et al. 2012; Zang 2020; Xu and Zang 2022). Since its formal proposal, the concept of PSESP has become a central focus of biodiversity conservation in China. Most PSESP are endemic to China and have important ecological, economic, and cultural values (Ren et al. 2012). Consequently, protecting PSESP is essential for maintaining ecological balance and preventing species extinction, with significance extending beyond China to global biodiversity conservation (Zhang et al. 2018).

Genetic diversity, a core component of biodiversity, underpins species' adaptive capacity. High genetic diversity enhances the survival ability of wild plants and reduces their extinction risk, making it possible to predict species fitness through the evaluation of genetic diversity (Hu et al. 2022; Wu et al. 2024). To understand the evolutionary and adaptive potential of populations, genetic differentiation and gene flow are key determinants. High gene flow facilitates genetic exchange among populations, reducing inbreeding and population differentiation (Waqar et al. 2021). In contrast, small plant populations are prone to genetic drift, which can lead to a rapid loss of genetic diversity and increased genetic differentiation among populations. Such processes can reduce population viability and adaptive potential, while high levels of genetic differentiation may result in outbreeding depression (Yang et al. 2022). Many factors influence plant genetic diversity, including seed dispersal, mating systems, life history, geographic distribution, and evolutionary history. Therefore, investigating the genetic composition of PSESP, clarifying their evolutionary history and population dynamics, and uncovering their genetic mechanisms for responding to environmental changes are essential to understand their endangerment mechanisms and implementing effective conservation strategies. These efforts are also significant components of conservation biology research (Xu and Zang 2022). However, despite their conservation importance, genomic‐scale datasets remain unavailable for many PSESP, limiting the precision of conservation management.

**FIGURE 1:**
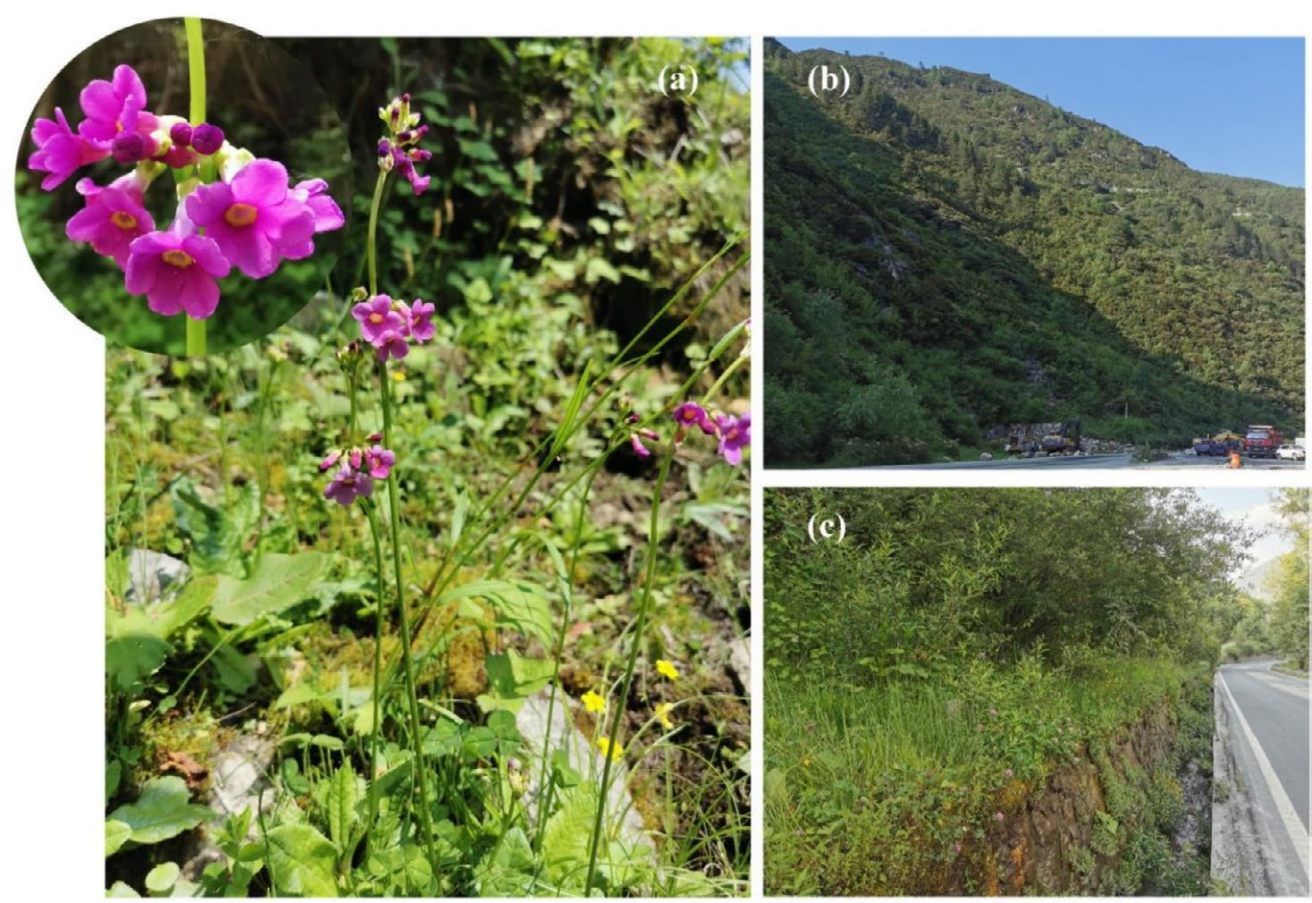
Field morphology and disturbed habitats of *Primula wilsonii* . (a) Flowering individual showing the characteristic whorled umbel inflorescences in its natural habitat. (b) Habitat degradation caused by road construction. (c) Population fragmentation by the road.

*Primula wilsonii* Dunn (Figure 1a) is a perennial herbaceous species belonging to the Section *Proliferae* of the genus *Primula*. It is characterized by multiple whorls of umbel inflorescences and exhibits the heterostylous breeding system that is common within the genus. The species is primarily distributed in the southwestern mountainous regions of China, where it inhabits moist slopes and stream banks at elevations of approximately 2000–3300 m. Owing to its fragmented habitat, restricted distribution, and small population size, *P. wilsonii* is classified as a plant species with an extremely small population (Sun et al. 2019). Like other *Primula* species, it has considerable ornamental value and certain medicinal uses. To date, research on *P. wilsonii* has remained limited. Its complete chloroplast genome has been sequenced, revealing a close phylogenetic relationship with species such as *P. anisodora* and *P. poissonii* (Xie et al. 2021, 2023). Additionally, comparative transcriptome studies have provided extensive transcript resources and molecular markers (EST SSR, single‐copy nuclear gene primers) and have estimated divergence time between *P. wilsonii* and *P. poissonii* at approximately 0.90 ± 0.57 Ma (early Middle Pleistocene), offering molecular evidence for rapid radiation and adaptive differentiation (Zhang et al. 2013). Despite these advances, no study to date has investigated the population genomics and conservation genetics of *P. wilsonii* using genome‐wide SNP data, leaving a critical gap in our understanding of its evolutionary history and genetic vulnerability. Barcoding studies have also assessed the discriminatory power and taxonomic significance of commonly used loci at both the section and genus‐wide level of *Primula* (Yan et al. 2011, 2015). However, information on the conservation genetics of *P. wilsonii* remains relatively limited, which hinders the development and implementation of viable long‐term conservation plans.

The emergence of next‐generation sequencing (NGS) technologies has profoundly reshaped the field of conservation genetics research (Hendricks et al. 2018; Cano et al. 2022; Yan et al. 2024). Previous research primarily relied on chloroplast DNA sequences, single‐copy nuclear genes, and microsatellite markers to investigate population structure, genetic diversity, and genetic characterization. As NGS has become more affordable over the past few decades, researchers have increasingly used Restriction site‐associated DNA sequencing (RAD‐seq) to provide sufficient resolution with rich single‐nucleotide polymorphisms (SNPs) (Li et al. 2023). Due to their abundance, stability, and suitability for automated analysis, SNPs are now the preferred molecular markers for intraspecific genomic variation (Shekhovtsov et al. 2022), and have been widely applied in plant population genetic studies (Ruang‐areerate et al. 2023; Yuan et al. 2023; Tang et al. 2024; Wu et al. 2024). For PSESP, genome‐wide SNP data are particularly valuable because they improve the accuracy of estimates of genetic diversity, population structure, and demographic history, thereby enabling more informed conservation strategies. Consequently, analyzing species' population history and adaptive evolution to uncover extinction risks of PSESP has become a conservation biology research hotspot (Xu and Zang 2022).

Here, we employed ddRAD‐seq to generate a high‐density panel of genome‐wide SNPs to investigate the genetic diversity, evolutionary history and population dynamics of *P. wilsonii* . Integrating with Species Distribution Models (SDMs), we aimed to reveal the genetic mechanisms underlying its response to environmental changes to provide scientific evidence for understanding its endangerment mechanisms and implementing corresponding conservation measures. We specifically incorporated both neutral demographic inference and ecological niche modeling to reconstruct historical dynamics and future habitat trends. The primary objectives of this study were to: (1) elucidate the genetic structure, genetic diversity, and genetic differentiation of the species through detailed analysis of population structure; (2) investigate the historical expansion patterns of *P. wilsonii* populations, thereby providing insights into the mechanisms underlying its endangerment; and (3) simulate the potential distribution patterns of the species across different time periods and assess the key environmental factors influencing its habitat suitability. This integrative framework provides a comprehensive basis for delineating conservation units and formulating species‐specific management strategies. Collectively, these analyses provide a robust scientific basis for the development of practical and effective conservation strategies for *P. wilsonii* .

## 2 Materials and Methods

### 2.1 Sample Collection and DNA Extraction

**FIGURE 2:**
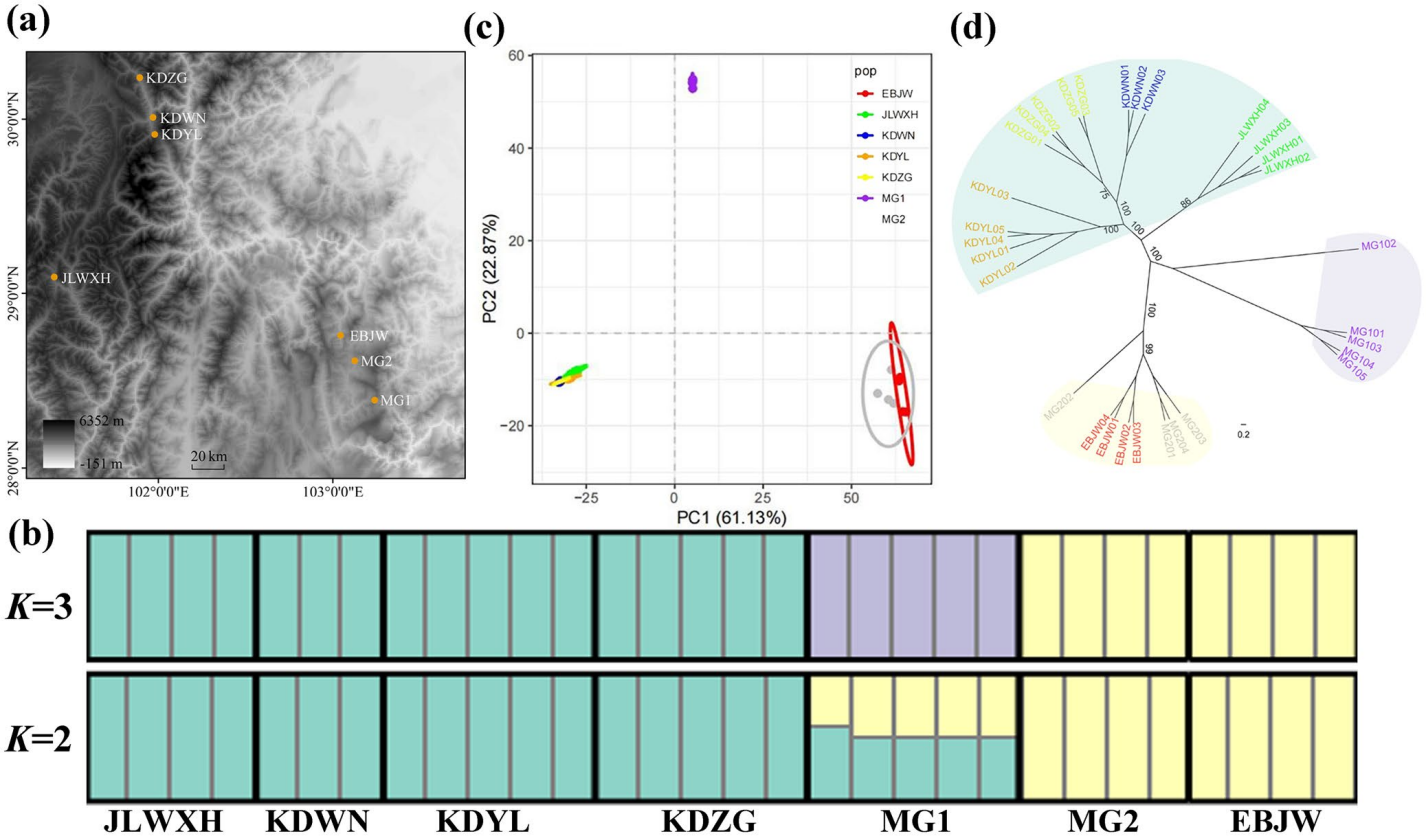
Field sampling and population genetic structure of *Primula wilsonii* . (a) Population sampling localities. (b) STRUCTURE analysis of the studied individuals in *P. wilsonii* populations for *K* = 2 and *K* = 3, the elevation of each colored bar represents the probability of assignment to the corresponding population. (c) Principal component analysis (PCA) with colors indicating each population and each individual shown as a square. (d) ML phylogenetic tree; colors are harmonized across panels (b–c).

In this study, *P. wilsonii* individuals were collected from Sichuan Province, and fresh leaves were preserved using silica gel drying. A total of 30 individuals were sampled from seven wild populations (Table 1, Figure 2a). Genomic DNA was extracted using a Tiangen DNA extraction kit. DNA concentration and purity were assessed using a NanoDrop spectrophotometer, and precise quantification was performed using a Qubit Fluorometer. Only samples that passed the quality control were used for subsequent library construction.

**TABLE 1:** Sampling information for populations of *Primula wilsonii* .

Collection location	Longitude	Latitude	Altitude	Population	Individuals
Wuxuhai, Jiulong, Sichuan, China	101°23′ 54.75″	29°5′ 31.15″	3325 m	JLWXH	4
Meigu, Sichuan, China	103°13′ 23.94″	28°23′ 59.62″	2314 m	MG1	5
Meigu, Sichuan, China	103°6′ 46.62″	28°37′ 22.91″	2697 m	MG2	4
Zhonggu, Kangding, Sichuan, China	101°53′ 5.24″	30°14′ 3 8.71″	3019 m	KDZG	5
Laoyulin, Kangding, Sichuan, China	101°57′ 50.84″	29°56′ 16.36″	3102 m	KDYL	5
Wannian, Kangding, Sichuan, China	101°57′ 2.03″	30°0′ 8.01″	2860 m	KDWN	3
Jiawa, Ebian, Sichuan, China	103°2′ 37.17″	28°46′ 2.95″	1932 m	EBJW	4

### 2.2 RAD‐Seq Libraries and SNP Calling

Quality‐controlled genomic DNA was double‐digested with *EcoRI* and *NlaIII*, ligated to barcode adapters, and subjected to size selection and PCR amplification to create ddRAD‐seq libraries targeting fragments of 300–500 bp. Then, 150 bp paired‐end sequencing was performed on the Illumina HiSeq PE150 platform at Genepioneer Bioinformatics Technology Co. Ltd. (Nanjing, China). Raw reads were filtered using default parameters of Trimmomatic v0.39 (Sewe et al. 2022). De novo assembly and SNP calling were conducted using the denovo_map.pl script in Stacks v2.65 (Catchen et al. 2013; Rochette et al. 2019) with parameters m = 3, M = 6, *n* = 6. Variant sites were extracted using the populations program. To ensure the quality of subsequent analyses, secondary filtering was performed in VCFtools v4.2 (Danecek et al. 2011), retaining only biallelic SNPs with a genotype call rate ≥ 80% (*max missing* = 0.8) and MAF ≥ 0.05. The max‐alleles parameter was set to 2 to restrict analyses to biallelic loci. To minimize linkage disequilibrium (LD), we applied the vcf_parser.py script using the center‐snp option, which retains only the SNP closest to the midpoint of each RAD locus. Because SNPs within a RAD tag are physically adjacent and likely linked, whereas different RAD loci are effectively independent, this locus‐based thinning approach effectively minimizes LD without relying on *r* ^2^ threshold or sliding‐window pruning, and is widely applied in RAD‐seq population genomic studies (Rochette et al. 2019). To exclude loci potentially influenced by natural selection and environmental pressures, outlier SNPs were detected using two complementary methods: BAYESCAN v.2.1 (Foll and Gaggiotti 2008) and the R package pcadapt v.4.3.2 (Luu et al. 2017). The BAYESCAN significance threshold was set at 0.05, and *pcadapt* identified outliers using a false discovery rate (FDR) of α = 0.05. SNPs identified as outliers by either program were removed from downstream population genetics analyses.

### 2.3 Phylogenetic Analysis and Population Genetic Structure Analysis

Phylogenetic relationships among the seven populations of *P. wilsonii* were inferred using a Maximum Likelihood (ML) method. SNP data were converted from VCF file to the PHYLIP format using the Python script vcf2phylip. The optimal nucleotide substitution model was selected using ModelFinder in IQ‐TREE (Kalyaanamoorthy et al. 2017). ML tree reconstruction was performed in RAxML with 1000 bootstrap replicates. The results were visualized using FigTree v1.4.4 software. For population genetic structure analyses, the SNP VCF file was converted to the SPID format using PGDSpider v2.1.1.5 (Lischer and Excoffier 2012). Genetic clustering was evaluated with Structure_threader v.1.3.4 (Pina‐Martins et al. 2017), conducting 20 independent runs for each *K* value from 1 to 7. Each run included 100,000 burn‐in steps and 200,000 Markov Chain Monte Carlo (MCMC) repetitions. The K value with the largest ΔK is considered the most likely number of populations. Finally, Principal component analysis (PCA) was performed using the R packages ggplot2, vcfR, and poppr, based on the first three principal components (PC1, PC2, PC3).

### 2.4 Genetic Diversity and Genetic Differentiation

Genetic diversity parameters, including percentage of polymorphism, observed heterozygosity (*Ho*), expected heterozygosity (*He*), nucleotide diversity (π), inbreeding coefficient (*F*_IS_), and genetic differentiation index (*F*_ST_), were calculated using the populations program of the Stacks software. Nucleotide diversity, defined as the average number of nucleotide differences per site between two randomly chosen DNA sequences, can be effectively used to assess the genetic diversity levels and evolutionary potential of different populations or species in combination with observed heterozygosity (Nei 1978).

### 2.5 Population Historical Dynamics Analysis

To investigate the population demographic history of *P. wilsonii* , we employed two complementary approaches for mutual validation operating at different temporal scales. SMC‐based methods such as STAIRWAY PLOT are primarily sensitive to recent demographic changes, whereas FASTSIMCOAL2 models the full site frequency spectrum (SFS) and can recover deeper historical events (Liu and Fu 2020; Excoffier et al. 2013). First, we applied the Sequentially Markovian Coalescent (SMC) method, implemented in the program STAIRWAY PLOT v2.0 (Liu and Fu 2020), to infer changes in effective population size (*Ne*) from the unfolded SFS (generation time (g) of 3 years for *P. wilsonii* and the mutation rate (μ) of 7 × 10^−9^), referencing *Arabidopsis thaliana* (Nordborg et al. 2005; Gossmann et al. 2012). Second, we conducted coalescent simulations in FASTSIMCOAL2 v2.6.0.2 software (Excoffier et al. 2013), based on unfolded joint site frequency spectra (u‐SFS) generated from neutral and unlinked SNPs. Five scenario models were tested: (a) constant size, (b) expansion, (c) expansion followed by contraction, (d) contraction, and (e) contraction followed by expansion. For each model, 100 independent replicates were run with 250,000 simulations per replicate, using the Expectation‐Conditional Maximization (ECM) algorithm for 60 optimization cycles and a convergence threshold of 0.001.

### 2.6 Species Distribution Model Analysis

The location information was retrieved from the Global Biodiversity Information Facility (GBIF, https://www.gbif.org), the National Specimen Information Infrastructure (NSII, http://www.nsii.org.cn), and our field survey. To mitigate spatial autocorrelation and prevent model overfitting, redundant records within a 1‐km radius were removed using the SDMToolbox in ArcGIS 10.8, ultimately retaining 40 sites for subsequent analysis. 19 bioclimatic variables for five periods, including the Last Glacial Maximum (LGM, ca. 22,000 years ago), Mid‐Holocene (MH, ca. 6000 years ago), current and future periods (2021–2040, 2081–2100), were obtained from the climate database (https://worldclim.org/) (Fick and Hijmans 2017). Future climate projections were based on the CMIP6 BCC‐CSM2‐MR global climate model under two Shared Socioeconomic Pathway scenarios: SSP1‐2.6 (low‐emission scenario) and SSP5‐8.5 (high‐emission scenario). After removing highly correlated bioclimatic variables with Pearson correlation coefficients greater than |0.8|, five variables including bio1, bio3, bio4, bio12, and bio15 were selected for subsequent analyses.

Species distribution models were constructed using the Biomod2 platform, which combines 11 modeling algorithms (Thuiller et al. 2009). Each model was executed following the described procedure for three times, and the accuracy of the 11 models was assessed using the Receiver Operating Characteristic curve (ROC) and the True Skill Statistics (TSS). Models with TSS greater than 0.7 were selected to reconstruct the ensemble model (EM) using EMwmean (Ensemble Model‐Weighted Mean) method (Powell‐Romero et al. 2023; Wang et al. 2025). Variable importance scores and response curves were generated to assess the contribution of each climatic factor. The ensemble model was used to predict the potential distribution areas of the species under past and current climatic conditions, as well as future climatic conditions. Habitat suitability was visualized in ArcGIS and classified into four categories: unsuitable (0–0.15), low suitability (0.15–0.50), medium suitability (0.50–0.75), and high suitability (0.75–1.00).

## 3 Results

### 3.1 Sequencing Data Characteristics

After quality control of the reduced‐representation genome sequencing data, a total of 41.38 Gb of high‐quality clean data was obtained. The statistical analysis results of the total read number, total base number, sequence length, GC content, Q20 pass rate, and Q30 pass rate of the paired‐end clean data for the 30 samples are shown in Table S1. The average Q20 proportion was 90.06% and the average Q30 proportion was 89.4%, indicating high sequencing quality suitable for subsequent analysis. Sequencing coverage of the reduced‐representation genome ranged from 9.5× to 16.7×, with an average coverage of 12.8×. A total of 1,140,991 loci were obtained, of which 99.8% had paired‐end overlap, with an average length of 237.22 bp. Stacks software initially detected 1,140,991 raw SNPs, and after quality filtering, 18,313 neutral and unlinked SNPs were retained for subsequent analysis.

### 3.2 Population Structure Analysis

Based on 18,313 neutral and unlinked SNPs, population genetic clustering analysis was performed on the seven populations using Structure_threader. The results indicated that *K* = 2 yielded the highest ΔK value, suggesting the strongest support for two major clusters. Population genetic structure diagrams for *K* = 2 and 3 are shown in Figure 2b. At *K* = 2, populations KDWN, KDYL, KDZG, and JLWXH clustered together, while EBJW and MG2 formed another group, with the ancestry of MG1 being in a mixed state. When *K* = 3, population MG1 had no mixed components and formed a distinct group, clearly separated from the other populations.

PCA further supported this pattern. The first two principal components PC1 and PC2 explained 61.13% and 22.87% of the total variance, respectively. As shown in Figure 2c, the first principal component PC1 separated the Kangding‐Jiulong (KDWN, KDYL, KDZG, JLWXH) from the Meigu and Ebian populations (MG1, MG2, EBJW), while PC2 further distinguished MG1 from the other populations. Overall, PCA divided the seven populations into three major clusters.

Phylogenetic relationships inferred using the Maximum Likelihood approach were consistent with both STRUCTURE and PCA results. The best‐fitting nucleotide substitution model was identified as GTR + γ using ModelFinder. The phylogenetic tree constructed using RAxML (Figure 2d) showed that JLWXH clustered with the three Kangding populations (KDWN, KDYL, KDZG), whereas EBJW clustered with MG2, both with high support values. Population MG1 was separated from the other populations.

### 3.3 Genetic Diversity and Genetic Differentiation

Genetic diversity estimates for the seven populations are summarized in Table 2. Both *Ho* and *He* were extremely low across all populations, with *Ho* ranging from 0.099729 to 0.099816 and *He* ranging from 0.00184 to 0.00271. Nucleotide diversity (π) ranged from 0.00176 to 0.00292, indicating overall low levels of nucleotide diversity. The highest nucleotide diversity was found in the Meigu population MG2 (π = 0.00292), while the three populations from Kangding exhibited relatively low genetic diversity levels, with the KDYL population showing the lowest value (π = 0.00163). *F*_IS_ values were close to zero, indicating that most populations were in Hardy–Weinberg equilibrium. The *F*_IS_ values for the MG1 and MG2 populations were positive, suggesting slight inbreeding, while the *F*_IS_ values for the other populations were negative, indicating slight outbreeding. Notably, both *He* (0.00184–0.00271) and π (0.00176–0.00292) are several orders of magnitude lower than those typically reported for outcrossing perennial plants, including many threatened species, highlighting the extremely depauperate genetic state of *P. wilsonii* and its long‐term small effective population size.

**TABLE 2:** Genetic diversity parameters within populations of *Primula wilsonii.* Observed heterozygosity (*Ho*) represents the proportion of heterozygous genotypes across polymorphic SNP sites, whereas expected heterozygosity (*He*) and nucleotide diversity (π) are allele‐frequency‐based measures of genetic diversity.

Pop ID	Percentage of Polymorphic Loci (P%)	*Ho*	*He*	π	*F*_*IS*_
JLWXH	0.387	0.00234	0.00163	0.00231	−0.00004
MG1	0.4762	0.00222	0.00191	0.00245	0.00048
MG2	0.51545	0.00271	0.00213	0.00292	0.00038
KDZG	0.30164	0.00193	0.00128	0.00176	−0.00027
KDYL	0.27436	0.00184	0.00117	0.00163	−0.00035
KDWN	0.27852	0.00198	0.00124	0.00183	−0.00023
EBJW	0.43283	0.00241	0.0018	0.00246	0.00009

Pairwise genetic differentiation further supported the three‐cluster structure (Table 3). Genetic differentiation was lowest within the Kangding‐Jiulong cluster, with the smallest *F*_ST_ observed between KDWN and KDYL (*F*_ST_ = 0.143). In contrast, differentiation between clusters was markedly higher. Populations from Ebian and Meigu (MG2) showed moderate differentiation from the Kangding‐Jiulong cluster, whereas MG1 exhibited extremely high differentiation from all other populations (*F*_ST_ > 0.7), consistent with its deep genetic isolation.

**TABLE 3:** The pairwise *F*_ST_ of seven populations of *Primula wilsonii* .

Cluster	Population	KDWN	KDYL	KDZG	JLWXH	EBJW	MG2	MG1
KD + JL	KDWN	—						
	KDYL	0.143144	—					
	KDZG	0.14758	0.20213	—				
	JLWXH	0.212768	0.24765	0.245392	—			
EB + MG2	EBJW	0.808031	0.820921	0.81622	0.784968	—		
	MG2	0.772163	0.786496	0.782758	0.751404	0.183594	—	
MG1	MG1	0.716603	0.734001	0.728617	0.751404	0.737508	0.711196	—

### 3.4 Population Historical Dynamics Analysis

**FIGURE 3:**
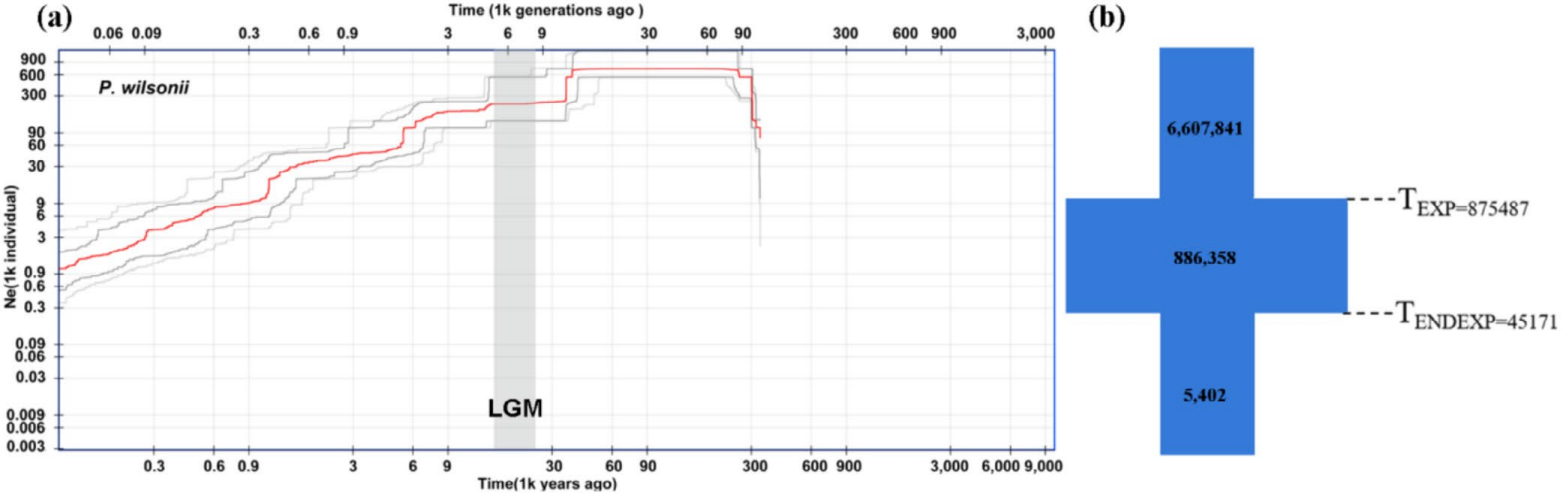
Demographic population history of *Primula wilsonii*. (a) Changes in effective population size (*Ne*) inferred using STAIRWAY PLOT v2.0 based on the unfolded site frequency spectrum. The Last Glacial Maximum (LGM; c. 26.5–19 kya) is highlighted in gray vertical bars. (b) Demographic scenarios modeled, using FASTSIMCOAL, with median times in generation (3 years per generation for *P. wilsonii* ), as well as estimates of effective population sizes.

Based on the analysis using STAIRWAY PLOT v2.0, the effective population size (*Ne*) of *P. wilsonii* began to decline around 40 ka, in the late LGM (10–70 ka), and has remained in a continuous contraction state (Figure 3a). Demographic history was inferred using the SMC‐based method implemented in STAIRWAY PLOT v2.0, which reconstructs changes in effective population size from the unfolded site‐frequency spectrum of multiple individuals. The model fit comparison from the FASTSIMCOAL2 identified the expansion–contraction model as the best‐supported scenario (model c; ΔAIC = 0; Figure 3b). The population history parameter assessment of the optimal model indicates that the population began to expand around 0.88 Ma and ended the expansion, starting to contract around 45 ka. Although the two approaches differ in temporal sensitivity, both consistently indicate long‐term population decline following an ancient expansion.

### 3.5 Species Distribution Model Analysis Results

**FIGURE 4:**
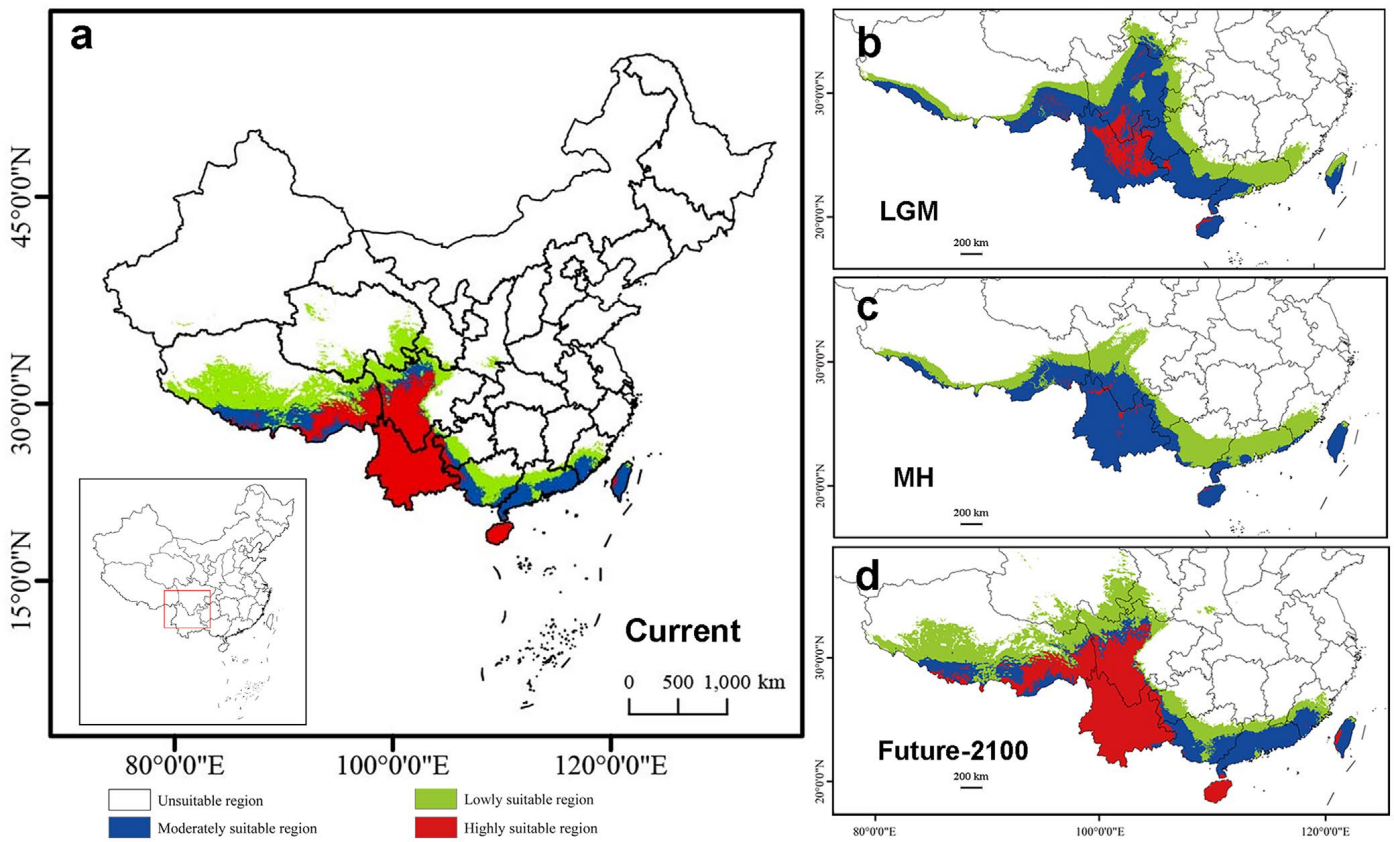
Habitat suitability of *Primula wilsonii* predicted by species distribution models (SDMs) for (a) current, (b) last Maximum Glacial (LGM), (c) mid‐Holocene (MH) and (d) the future‐2100 (SSP1‐2.6).

Models with TSS value greater than 0.7 were retained to construct the ensemble model using EMwmean method. All 11 models met this criterion (Table S2), yielding an ensemble model with excellent performance (AUC = 1.0; TSS = 1.0). Among the five environmental variables used in the modeling, bio4 (temperature seasonality variance) had the highest importance score of 50.86% (Table S3), indicating its significant influence on the model. The other environmental variables had importance scores below 10%. The response curve for bio4 showed a unimodal pattern, indicating highest habitat suitability within a certain intermediate range of temperature seasonality variance, with suitability declining toward both lower and higher extremes. The environmental variables bio3 and bio12 showed positive correlations with the distribution probability of *P. wilsonii* , while bio1 and bio15 showed no consistent relationships (Figure S1). According to the ensemble predictions (Figure 4), current high‐suitability habitats of *P. wilsonii* are mainly distributed in southwestern China, particularly in high‐altitude regions of Yunnan, Sichuan, and Guizhou, with additional suitable areas in Hainan and Taiwan. The simulation results of the ensemble model based on Biomod2 for different climate scenarios show that from the LGM to the MH, the suitable habitat area contracted to some extent. From the MH to the present, there was a trend of southward expansion of suitable habitat areas. Under future climate‐warming scenario, suitable habitats are mainly concentrated in the Hengduan Mountains, with little change compared to the current suitable distribution areas. This indicates the persistence of climatically stable refugial regions in southern Yunnan and Sichuan. Projections under the high‐emission scenario (SSP5‐8.5; Figure S2) were highly consistent with those under SSP1‐2.6.

## 4 Discussion

### 4.1 Reliability and Applications of SNP Data

The biallelic nature, genome‐wide coverage, and high reproducibility of single‐nucleotide polymorphisms (SNPs) make them the primary markers in population genetics and conservation genomics (Allendorf et al. 2010; Rellstab et al. 2015; Kardos et al. 2021; Hoelzel 2023; Miller et al. 2023; Aitken and Bemmels 2024), and particularly well suited for genetic studies of endangered species in which sample sizes are inevitably limited. For instance, reduced‐representation genomic studies of *Pinus torreyana* have leveraged thousands of SNPs to elucidate island–mainland population history and adaptive differentiation, thereby weighing the trade‐offs of genetic rescue and illustrating the value of SNPs for separating neutral from selective signals and for evaluating conservation interventions (Di Santo et al. 2022). Similarly, whole‐genome resequencing of the critically endangered *Magnolia sinica* revealed two demographic bottlenecks, providing an empirical basis for partitioning PSESP populations and managing genetic load (Cai et al. 2024). A comparative study of two rare oaks, *Quercus acerifoli and Q. boyntonii*, further demonstrated that SNPs offer higher resolution than microsatellites in individual assignment and population structure resolution, enabling optimized ex situ sampling strategies and evaluation workflows (Koontz et al. 2024). Accordingly, SNPs are capable of not only inferring evolutionary processes, but also serving as decision‐making tools to safeguard genetic diversity and the long‐term adaptive potential of species (Kardos et al. 2021; Dussex et al. 2023). These indicators collectively confirm that the SNP dataset is sufficiently robust for high‐confidence inference of population structure, diversity, and demographic history.

The reduced‐representation genome data generated in this study meet the conventional thresholds for population‐level genomic inference, including high base‐calling quality (Q20 ≈ 90.06%, Q30 ≈ 89.4%), sufficient sequencing depth per sample (9.5×−16.7×; mean 12.8×), extensive number of loci (1,140,991 before filtering; 18,313 neutral unlinked SNPs after filtering), and a high paired‐end overlap proportion (99.8%). Compared with congeneric studies, our data scale is comparable to that of *Primula tibetica* and *P. fasciculata* in RAD‐seq (thousands to tens of thousands of SNPs, moderate sequencing depth), which is sufficient to support the analysis of population genetic structure and historical dynamics (Ren et al. 2017, 2020). Relative to some extremely small populations of wild plants or endangered plants (such as *Cypripedium macranthos*, *Anemone shikokiana*, *Pinus torreyana* , *Magnolia sinica*), the scale and coverage depth of our neutral unlinked loci also fall within the common analytical range, and paired‐end sequencing and overlap help to reduce misgenotyping and linkage artifacts, and improve genotype consistency (Rochette et al. 2019; Di Santo et al. 2022; Wu et al. 2023; An et al. 2024; Cai et al. 2024). Although some populations were represented by small sample sizes, this limitation is partly mitigated by the use of genome‐wide ddRAD‐seq data. Simulation studies have shown that thousands of independent SNPs can yield reliable estimates of population differentiation (*F*_ST_) even with only 3–5 individuals per population (Willing et al. 2012). Importantly, the concordant signals from STRUCTURE, PCA, *F*_ST_, and demographic analyses indicate that the high information content of the SNP dataset compensates for sampling limitations. Therefore, the data quality and locus design here provide a reliable evidentiary basis for subsequent management and conservation‐intervention assessments.

### 4.2 Causes of Low Within‐Population Genetic Diversity

Genetic diversity constitutes the fundamental raw material for adaptation, strongly influencing fitness, evolutionary potential, and long‐term persistence. In general, reduced genetic diversity correlates positively with constrained adaptive potential and elevated extinction risk (Frankham 2005; Kardos et al. 2021). Multiple factors shape genetic diversity, including effective population size (*Ne*) and demographic history. For example, long‐term small *Ne* and bottlenecks accelerate genetic drift and the loss of rare alleles, resulting in extremely low heterozygosity and high *F*_ST_, as documented in *Trillium govanianum* (Islam et al. 2023). Mating system and life history also affect genetic diversity; the long‐lived, predominantly outcrossing woody plant *Rhododendron protistum* var. *giganteum* maintains relatively high genetic diversity despite having only two extant populations (Wu et al. 2014). Landscape connectivity and land‐use legacies also play a critical role; reduced connectivity can weaken gene flow and erode local diversity, as demonstrated in *Primula veris* (Reinula et al. 2024). Under more favorable mating systems and connected landscapes, some endangered/endemic plants can still maintain moderate to high diversity within populations such as *Paeonia decomposita* (Wang 2020).

Notably, the genetic diversity of *P. wilsonii* is orders of magnitude lower than that reported for other *Primula* species based on genome‐wide SNP data (e.g., π ≈ 0.003~0.007 in *P. tibetica* and *P. fasciculata* ; Ren et al. 2017, 2020), and far below values observed in many endangered plants assessed using SSRs (e.g., *He* ≈ 0.02~0.05 in *Trillium govanianum*; Islam et al. 2023). Despite these extremely low levels of *He* and π, *Ho* was relatively uniform across populations. Such patterns of *Ho*–*He* decoupling have been reported in small or isolated plant populations genotyped with genome‐wide SNPs (Ren et al. 2017; Wu et al. 2023). This apparent discrepancy likely reflects the combined effects of long‐term small *Ne* and persistent topography‐driven isolation, under which genetic drift rapidly removes rare alleles while remaining polymorphisms persist at intermediate frequencies. Deep‐cut canyons and short‐distance seed and pollen dispersal jointly restrict allele exchange, leading to a significant decline in diversity. Field observations indicate that several populations of Kangding have been damaged by road construction (Figure 1b,c), leaving very few individuals and likely exacerbating diversity loss. Within the distributional range, engineering activities such as road cutting and landslides, as well as natural disturbances, can directly compress habitable patches, reduce patch quality, and raise resistance among patches, thereby shortening effective pollinator and seed dispersal distances, reducing gene flow and further lowering *Ne*, and ultimately producing low within‐population diversity and elevated among‐population differentiation (Forman and Alexander 1998; Aguilar et al. 2006; Vranckx et al. 2012). Similar landscape legacy effects have been documented in *Primula veris* (Reinula et al. 2024), and such pressures are expected to intensify in mountain systems worldwide under ongoing infrastructure expansion and geohazards exposure (Dragonetti et al. 2024). Taken together, demographic inference and diversity estimates converge on a scenario in which prolonged small *Ne* and restricted connectivity have driven extreme genetic erosion in *P. wilsonii* .

### 4.3 The Patterns and Causes of Population Genetic Structure and Differentiation

Population genetic structure and differentiation emerge from the long‐term coupling of gene flow, genetic drift, and natural selection under specific geological, climatic, and landscape contexts (Slatkin 1987; Hewitt 2000; Sexton et al. 2014; Wang and Bradburd 2014). In this study, the population genetic structure of *P. wilsonii* exhibited a significant hierarchical structure with three clusters. STRUCTURE analyses identified two major groups at K = 2 (Kangding + Jiulong vs. Ebian + Meigu), whereas at K = 3, MG1 formed a distinct and isolated cluster. This three‐cluster pattern was consistently supported by PCA (PC1 = 61.13%, PC2 = 22.87%) and the phylogenetic analyses. Moreover, MG1 had pairwise *F*_ST_ > 0.7 with all other populations, while KDWN–KDYL had the lowest *F*_ST_ (0.143). This spatial pattern reflects a classic “sky island” configuration shaped by strong mountain–valley barriers and limited dispersal in the Hengduan region. Kangding and Jiulong populations occur within a relatively continuous high mountain–canyon system, whereas Ebian and Meigu occupy a more connected transitional zone at the basin margin. In contrast, MG1 appears to be subject to long‐term geographic isolation and reduced effective population size. Complex topography and Quaternary climate oscillations are the principal drivers in the formation of deep differentiation and hierarchical structure in this region. For example, the congeners *P. fasciculata* also show multiple genetic clusters and a glacially driven history of differentiation–expansion/bottlenecks (Ren et al. 2020), and *P. tibetica* reveals multiple lineages and refugia (Ren et al. 2017).

Comparable patterns of low within‐population diversity and high among‐population differentiation are also observed in other endangered plants. For instance, *Trillium govanianum* shows extremely low heterozygosity and high differentiation between populations based on SSR markers (*F*_ST_ ≈ 0.707), reflecting bottlenecks and restricted gene flow (Islam et al. 2023). The orchid *Cypripedium macranthos* likewise exhibits low within‐population variation and limited gene flow based on SNPs (Wu et al. 2023). In contrast, species with more favorable mating systems or high landscape connectivity, such as *Paeonia decomposita*, can maintain substantially higher genetic diversity (*He* = 0.49; Wang 2020). Taken together, the low‐diversity and high‐differentiation pattern in *P. wilsonii* likely reflects the combined effects of habitat degradation, long‐term small *Ne*, and topography‐driven isolation, implying limited evolutionary response space under future environmental disturbance. The concordance across multiple analytical frameworks provides strong genetic justification for delineating at least three independent management units. Subsequent management should focus on increasing or maintaining *Ne* and improving functional connectivity. Based on the three‐cluster pattern and the extremely deep differentiation of MG1, at least three independent management units (KD + JL, EB + MG2, MG1) should be delineated at the geographic unit scale. Within these units, priority should be given to maintaining or enhancing functional connectivity and effective population size to prevent further loss of diversity and support long‐term adaptive potential (Kardos et al. 2021; Dussex et al. 2023; Reinula et al. 2024).

### 4.4 Demographic History and Responses to Future Climate Change

Demographic reconstructions using FASTSIMCOAL2 inferred a long‐term expansion beginning around 0.88 Ma followed by a continuous contraction since about 45 ka. The STAIRWAY PLOT captured only the more recent decline starting around 40 ka. This difference is expected because STAIRWAY PLOT is primarily sensitive to recent SFS signals and has limited power to detect ancient demographic events, whereas FASTSIMCOAL2 fits the full SFS under explicit demographic models and therefore recovers broader‐scale historical changes. Thus, the two approaches are complementary rather than contradictory, capturing demographic processes operating at different temporal scales. Species distribution models further demonstrated that suitable habitat contracted from the LGM to the MH and subsequently expanded southward to the present. Under future warming scenarios, the high‐suitability area remains largely confined to the Hengduan Mountains and exhibits a unimodal response to temperature seasonality (bio4). Importantly, model projections indicate that these high‐suitability areas are largely stable in location rather than undergoing pronounced spatial shifts within the Hengduan Mountains, suggesting the persistence of core refugial patches under both low‐ and high‐emission scenarios. These results are highly consistent with the climate‐forcing pattern that followed the Mid‐Pleistocene Transition (MPT), characterized by increased glacial amplitude and a shift in glacial–interglacial rhythms from 41 kyr to 100 kyr (Herbert 2023). They also align with the habitat compression caused by the combined effects of ice sheets, greenhouse gases, and albedo at the LGM (Shi et al. 2023). From a paleobiogeographic standpoint, the extant genetic architecture of *P. wilsonii* reflects the imprint of Quaternary climatic fluctuations interacting with the complex mountain‐deep valley‐large river system of the Hengduan Mountains, which provides strong resistance and high micro‐topographic heterogeneity and a “sky island” pattern of hierarchical and deep lineage isolation (Hewitt 2000). This mechanism has been documented in *P. fasciculata* and *P. tibetica* from the same region (Ren et al. 2017, 2020), reinforcing the role of Quaternary legacies in shaping contemporary genetic structure.

In recent years, studies utilizing whole‐genome sequencing or high‐throughput markers have also revealed recurrent bottlenecks and regional differentiation under the coupling of Quaternary climate and topography in several PSESP and endangered plant species. For instance, the whole‐genome resequencing of the critically endangered *Artocarpus nanchuanensis* detected three bottlenecks and low genetic diversity, and inferred a southward shift of its suitable habitat range based on species distribution modeling (SDM) (Xia et al. 2023). The conservation genomics study of *Thuja sutchuenensis*, an endangered species in the Dabashan Mountains, found low genetic diversity but also low genetic load, suggesting a “vulnerable‐yet‐restorable” window under long‐term small effective population size (*Ne*) and topographic isolation (Tao et al. 2024). The population study of the “living fossil” *Tetracentron sinense* revealed a tripartite genetic structure and bottleneck, reflecting the combined effects of glacial legacy and human disturbance (Tian et al. 2025; whole‐genome work is also in progress). In the same region, phylogeography and SDMs of *Pleurospermum foetens* support lineage–environment coupling between “sky islands” and glacial–interglacial driven differentiation/expansion (Yu et al. 2024). By contrast, conservation genomics of *Acer yangbiense* showed low diversity and historical decline, revealed coexistence of population structure and relatively frequent gene flow across different loci, implying that species‐specific dispersal kernels and human disturbance histories can substantially modulate historical trajectories (Ma et al. 2022). Collectively, these examples all demonstrate that under combined effects of climate forcing, topographic resistance, dispersal nuclei, and human disturbance, the contraction, persistence, and marginal expansion of PSESP exhibit cross‐species commonalities.

Mountain plants typically exhibit two primary responses to ongoing warming and increasing climatic extremes. One involves local micro‐migration and core habitat stabilization via micro‐refuges and isothermal corridors, which can help maintain effective population sizes and genetic variation over short to medium timescales. The other involves restricted clustering and gene flow caused by landscape fragmentation and reduced connectivity, potentially further strengthening spatial structure and phylogenetic differentiation (Wang and Bradburd 2014; Dragonetti et al. 2024). Complex topography provides micro‐refuges and short‐distance isothermal corridors, enabling populations to buffer climatic fluctuations through local micro‐migration in terms of altitude and slope orientation. However, deep canyons and discontinuous high‐elevation plateaus constrain long‐distance dispersal and gene flow across mountain ranges, thus maintaining or strengthening spatial genetic structure (Malanson et al. 2024). The unimodal response of *P. wilsonii* to bio4 indicates a narrow niche centered on intermediate seasonality, explaining why future high‐suitability areas remain largely localized in the Hengduan and facilitate refugial persistence. Consistent with the southward expansion of potential suitability detected here, other alpine–canyon taxa frequently exhibit a coupled pattern of microrefugial persistence and interglacial edge expansion (Ren et al. 2017, 2020), as demonstrated by the phlogeography of the “sky‐island” species *Pleurospermum foetens* (Yu et al. 2024). Overall, the evolutionary history and climatic response of *P. wilsonii* reflect dual coupling between climatic forcing (MPT/LGM) and topographic resistance. At the glacial–interglacial timescales, expansion and contraction reshaped effective size, whereas at the landscape scales, valleys and drainage divides blocked long‐distance gene flow. This framework not only explains the observed three‐cluster hierarchical structure and deep lineage isolation in *P. wilsonii* , but also suggests that under future warming, the species is likely to rely on in situ micro‐shifts and core‐habitat stability, rather than large‐scale outward expansion or cross‐range amalgamation (Herbert 2023; Shi et al. 2023; Malanson et al. 2024).

## Author Contributions

**Yanping Xie:** conceptualization (supporting), data curation (lead), funding acquisition (lead), methodology (supporting), resources (equal), software (supporting), writing – original draft (lead), writing – review and editing (equal). **Xin Jin:** data curation (supporting), resources (equal), software (lead), writing – original draft (supporting), writing – review and editing (equal). **Ganggang Yang:** data curation (supporting), resources (equal), software (supporting), writing – review and editing (equal). **Dongting Lu:** data curation (supporting), resources (equal), software (supporting), writing – review and editing (equal). **Chan Zhang:** data curation (supporting), funding acquisition (supporting), resources (equal), writing – review and editing (equal). **Xingwang Zhang:** conceptualization (lead), resources (equal), writing – original draft (supporting), writing – review and editing (equal). **Xianfeng Jiang:** funding acquisition (supporting), methodology (lead), resources (equal), writing – original draft (supporting), writing – review and editing (equal).

## Funding

This work was supported by National Natural Science Foundation of China (Grants 32100169, 32271604, and 32460335) and Natural Science Foundation of Anhui Province (Grant 2108085QC104).

## Conflicts of Interest

The authors declare no conflicts of interest.

## Supporting information


**Table S1:** dd‐RAD tags sequencing of *Primula wilsonii* .
**Table S2:** The TSS and AUC values of the 11 models.
**Table S3:** Ranking of environmental variable importance.
**Figure S1:** Response curves of the five climatic factors.
**Figure S2:** Habitat suitability of *Primula wilsonii* predicted by species distribution models (SDMs) for the future. (a) 2040‐SSP1‐2.6. (b) 2040‐SSP5‐8.5. (c) 2100‐SSP1‐2.6. (d) 2100‐SSP5‐8.5.

## Data Availability

The sequencing reads used in this study are deposited in Sequence Read Archive (SRA) of GenBank under the accession number PRJNA1344917 (https://www.ncbi.nlm.nih.gov/sra/PRJNA1344917).
