# Supplementary material for: Genome‐Wide SNP Data and Species Distribution Modeling Reveal Population Structure and Conservation Implications of Primula wilsonii (Primulaceae)
Source: Ecol Evol. 2026 Jan 5;16(1):e72884. doi: 10.1002/ece3.72884 (PMC12771685; doi:10.1002/ece3.72884)
Supplement: Supplementary file 1 — Table S1: dd‐RAD tags sequencing of Primula wilsonii . Table S2: The TSS and AUC values of the 11 models. Table S3: Ranking of environmental variable importance. Figure S1: Response curves of the five climatic factors. Figure S2: Habitat suitability of Primula wilsonii predicted by species distribution models (SDMs) for the future. (a) 2040‐SSP1‐2.6. (b) 2040‐SSP5‐8.5. (c) 2100‐SSP1‐2.6. (d) 2100‐SSP5‐8.5. [file ECE3-16-e72884-s001.docx]

Appendix Table T1 dd-RAD tags sequencing of *Primula wilsonii*

| **Individuals** | **Reads numbers** | **base numbers** | **GC（%）** | **Q30（%）** | **Q20（%）** |
| --- | --- | --- | --- | --- | --- |
| JLWXH01 | 3,586,938 | 1,033,038,144 | 37.08 | 89.29 | 95.91 |
| JLWXH02 | 4,001,287 | 1,152,370,656 | 37.37 | 88.83 | 95.7 |
| JLWXH03 | 5,111,551 | 1,472,126,688 | 37.02 | 89.07 | 95.8 |
| JLWXH04 | 4,122,181 | 1,187,188,128 | 37.19 | 89.07 | 95.81 |
| MG101 | 4,757,486 | 1,370,155,968 | 37.36 | 89.91 | 96.27 |
| MG102 | 6,904,825 | 1,988,589,600 | 37.14 | 90.07 | 96.36 |
| MG103 | 5,529,880 | 1,592,605,440 | 37.32 | 89.63 | 96.16 |
| MG104 | 6,814,380 | 1,962,541,440 | 37.04 | 89.85 | 96.25 |
| MG105 | 5,222,471 | 1,504,071,648 | 37.3 | 89.78 | 96.22 |
| MG201 | 5,465,240 | 1,573,989,120 | 37.28 | 89.94 | 96.28 |
| MG202 | 4,677,413 | 1,347,094,944 | 37.41 | 89.89 | 96.27 |
| MG203 | 4,558,071 | 1,312,724,448 | 37.53 | 89.94 | 96.29 |
| MG204 | 3,998,418 | 1,151,544,384 | 37.35 | 89.17 | 95.96 |
| KDZG01 | 5,025,827 | 1,447,438,176 | 37.07 | 90.19 | 96.44 |
| KDZG02 | 4,547,659 | 1,309,725,792 | 37.17 | 86.65 | 94.85 |
| KDZG03 | 5,595,851 | 1,611,605,088 | 37.03 | 89.95 | 96.23 |
| KDZG04 | 3,916,697 | 1,128,008,736 | 37.01 | 89.46 | 96.08 |
| KDZG05 | 5,846,996 | 1,683,934,848 | 37.26 | 89.76 | 96.15 |
| KDYL01 | 3,721,113 | 1,071,680,544 | 36.61 | 89.09 | 95.95 |
| KDYL02 | 4,997,811 | 1,439,369,568 | 37.07 | 89.34 | 96.03 |
| KDYL03 | 5,089,758 | 1,465,850,304 | 36.87 | 89.32 | 96.04 |
| KDYL04 | 3,773,499 | 1,086,767,712 | 37.69 | 89.58 | 96.14 |
| KDWN01 | 5,163,803 | 1,487,175,264 | 37.27 | 88.32 | 95.52 |
| KDWN02 | 6,237,254 | 1,796,329,152 | 36.95 | 89.51 | 96.12 |
| KDWN03 | 5,076,267 | 1,461,964,896 | 37.07 | 89.5 | 96.11 |
| EBJW01 | 4,460,695 | 1,284,680,160 | 37.04 | 89.54 | 96.14 |
| EBJW02 | 7,495,674 | 2,158,754,112 | 37.24 | 89.22 | 95.99 |
| EBJW03 | 6,908,762 | 1,989,723,456 | 37.01 | 89.31 | 96.03 |
| EBJW04 | 4,433,958 | 1,276,979,904 | 36.77 | 89.4 | 96.07 |
| **Average** | 5,033,712 | 1,449,709,190 | 37.14 | 89.4 | 96.04 |

Appendix Table T2 The TSS and AUC values of the 11 models

| **Ranking** | **Models** | **TSS** | **AUC** |
| --- | --- | --- | --- |
| 1 | RF | 1 | 1 |
| 2 | XGBOOST | 1 | 1 |
| 3 | MARS | 0.985 | 0.999 |
| 4 | GLM | 0.985 | 0.998 |
| 5 | ANN | 0.985 | 0.992 |
| 6 | GBM | 0.978 | 0.999 |
| 7 | FDA | 0.974 | 0.99 |
| 8 | GAM | 0.944 | 0.993 |
| 9 | CTA | 0.924 | 0.962 |
| 10 | MAXENT | 0.914 | 0.957 |
| 11 | SRE | 0.733 | 0.867 |
|  | EMwmean | 1 | 1 |

Appendix Table T3 Ranking of environmental variable importance

| **Environmental variables** | **Importance score（%）** | **Ranking** |
| --- | --- | --- |
| bio4 (Temperature Seasonality) | 50.86 | 1 |
| bio3 ( Isothermality) | 4.51 | 2 |
| bio12 (Annual Precipitation) | 2.03 | 3 |
| bio1 (Annual Mean Temperature) | 0.1 | 4 |
| bio15 (Precipitation Seasonality) | 0.08 | 5 |


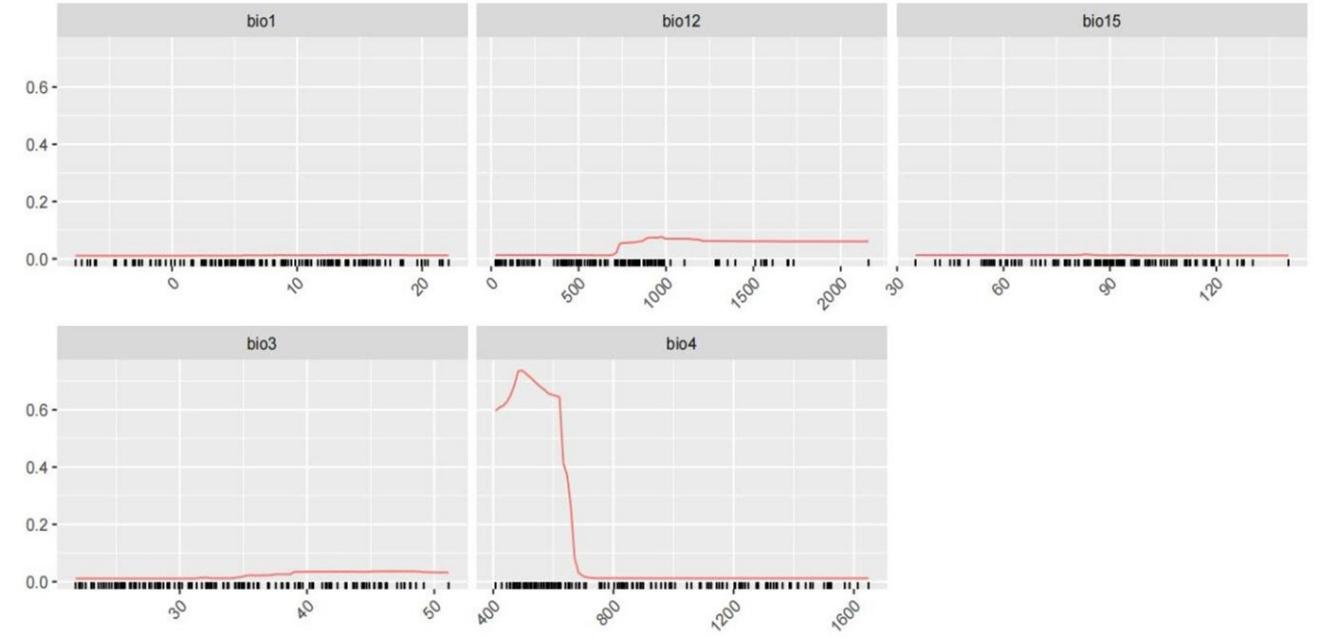


Appendix Figure S1. Response curves of the five climatic factors


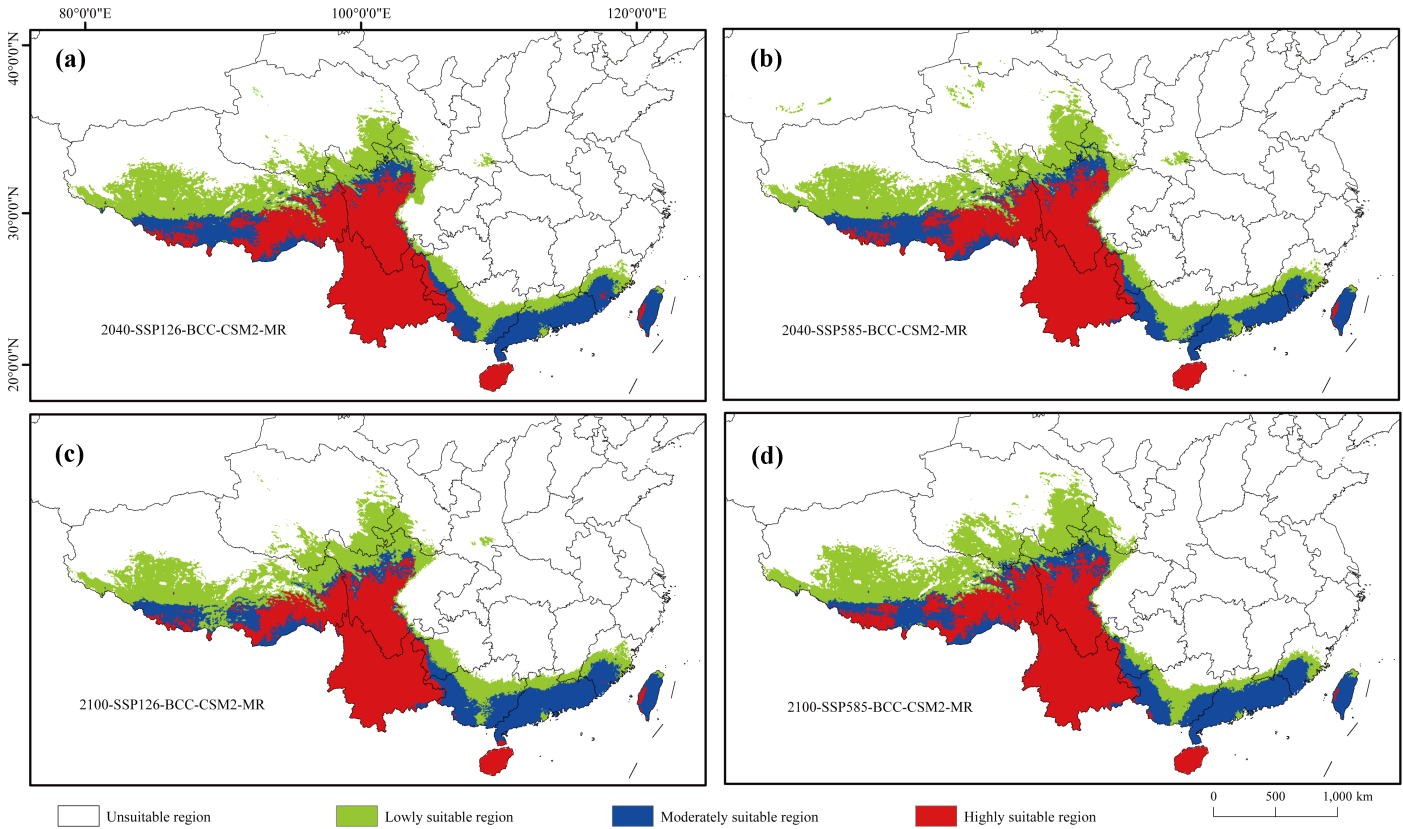


Appendix Figure S2. Habitat suitability of *Primula wilsonii* predicted by species distribution models (SDMs) for the future. (a) 2040-SSP1-2.6. (b) 2040-SSP5-8.5. (c)2100-SSP1-2.6. (d) 2100-SSP5-8.5.
